# Computation strategies and clinical applications in neoantigen discovery towards precision cancer immunotherapy

**DOI:** 10.1186/s40364-025-00808-9

**Published:** 2025-07-09

**Authors:** Zhenchang Wang, Yu Gu, Xiao Sun, Hao Huang

**Affiliations:** Institute of Microphysiological Systems, State Key Laboratory of Digital Medical Engineering, School of Biological Science and Medical Engineering, Southeast University, Nanjing, China

**Keywords:** Neoantigen discovery, Cancer immunotherapy, Artificial intelligence

## Abstract

Neoantigens, which are tumor-specific peptides generated by malignant cells, can be presented to T cells to elicit immune responses. Owing to their tumor-specific properties, neoantigens have emerged as one of the most promising biomarkers and targets for cancer immunotherapy. Previous studies have demonstrated their capacity to mediate tumor-specific immune responses in targeting and eliminating tumor cells while preserving normal cellular function. Driven by advancements in high-throughput sequencing technologies, mass spectrometry, and artificial intelligence, researchers have developed a growing interest in establishing more accurate neoantigen prediction algorithms. Here, we presented a comprehensive review of integrated neoantigen prediction algorithms, encompassing task definition, theoretical developments, benchmark datasets, cutting-edge applications, and future research directions. We systematically evaluated recent advancements in neoantigen source characterization and prediction algorithms, with particular emphasis on innovative methods for HLA-peptide binding and TCR recognition developed. Additionally, we explored the cutting-edge applications of neoantigens in personalized cancer vaccine design and adoptive cell therapies. We delineated potential research directions and the future prospects for neoantigen-based therapies, including integrating multi-omics data to discover universal neoantigens, addressing algorithmic generalization challenges and diversifying neoantigen validation methods.

## Introduction

Benefiting from the absence of adverse effects typically associated with conventional therapies like radiotherapy and chemotherapy, immunotherapy has emerged as a promising direction for breakthroughs in cancer treatment. Immune Checkpoint Inhibitors (ICIs) [1], Adoptive Cell Therapy (ACT) [2] and Cancer Vaccines (CVs) are representative techniques within the landscape of immunotherapy [3]. Despite numerous research outcomes in the aforementioned research directions, the number of results that have successfully transitioned into clinical phases is relatively limited. A critical factor contributing to this scarcity is the absence of suitable therapeutic targets. The efficacy of cancer immunotherapy critically depends on ensuring that therapeutic targets can be precisely submitted to specific T cells [4]. An ideal therapeutic target should be exclusively in cancer tissues and not expressed in healthy tissues.

Owing to their formation during carcinogenesis and strong immunogenicity, tumor antigens have been extensively discussed as an appropriate candidate target. Tumor antigens are peptides derived from newly synthesized protein molecules during carcinogenesis and can elicit a specific immune response from the immune system. Based on the degree of specificity and the differences in sources, tumor antigens can be categorized into cancer-testis antigens (CTAs) [5], virus-associated antigens, tumor-associated antigens (TAAs) and tumor-specific antigens (TSAs) [6]. CTAs are primarily localized in immune-privileged sites like the testes and placenta, and their limited expression in only a fraction of tumors limits their applicability as targets for immunotherapy. TAAs are antigens commonly found in both normal and tumor cells, yet exhibit a significantly higher level of expression in tumor cells. TAAs were initially employed as targets for immunotherapy. However, their low expression in normal cells induces central immune tolerance, thereby resulting in a weak immune response. Furthermore, the expression levels of TAAs are inconsistent among different patients even with the same type of cancer [7]. The existence of the aforementioned reasons has resulted in the failure of targeting TAAs to achieve the anticipated therapeutic outcomes in cancer treatment.

Compared to TAAs, TSAs exclusively expressed in tumor cells, better align with the individualized demands of immunotherapy and have thus garnered increasing attention in recent years [8]. TSAs emerge as a consequence of gene somatic mutations, RNA dysregulation and protein modifications during oncogenesis. When neoantigens serve as targets, their derived peptides are presented by human leukocyte antigen (HLA) molecules following intracellular processing, subsequently eliciting HLA-restricted T-cell immune responses. When neoantigens serve as targets, they can be presented by major histocompatibility complexes (MHC) molecules after expression, subsequently triggering an immune response by T cells. TSAs are not expressed in normal cells and are thus called neoantigens, thereby exhibiting more pronounced tumor specificity. The emergence and optimization of advanced sequencing techniques, including next-generation sequencing (NGS), have significantly heightened the focus on neoantigen research. Furthermore, as targets for immunotherapy, neoantigens can sustain immune responses, offering the potential for establishing long-term immunotherapeutic efficacy [9].

In this review, we will discuss how recent advances in neoantigens enriched the research on cancer immunotherapy. We will further summarize the technical features of the state-of-the-art prediction algorithms, analyzing their respective advantages and limitations of current methods, while highlighting recent breakthroughs in neoantigen-targeted cancer immunotherapy. Furthermore, we will propose challenges and opportunities to broaden the applicability of neoantigen across diverse clinical contexts, enhancing the generalizability of prediction models, and optimizing the viability and accuracy of immunogenicity verification protocols.

## Sources of neoantigens

### Somatic mutations

**Fig. 1:**
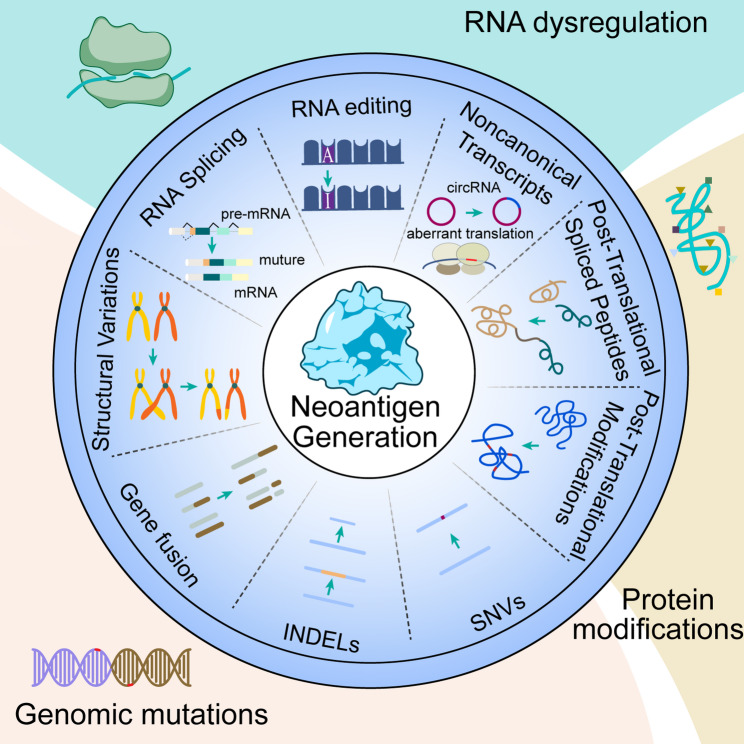
Sources of neoantigen generation. Neoantigens can be classified according to their origin as caused by variations in genomic level, transcriptomic level and protein level

Somatic mutations represent the primary drivers of neoantigen generation, encompassing single nucleotide variants (SNVs), insertions and deletions (INDELs), gene fusions and structural variations (See Fig. 1). SNVs and INDELs are the most extensively investigated mutation types and directly contribute to the calculation of tumor mutation burden (TMB). Although they occur in virtually all tumors, the total number of these mutations defines the TMB status of a tumor. Compared to SNVs and INDELs, neoantigens driven by gene fusions and structural variations were more prevalent in cancers with low mutation burden and reduced immune infiltration. Additionally, neoantigens generated by gene fusions had been associated with microsatellite-stable tumors [10, 11].

### Single nucleotide variations

SNVs are a specific form of nonsynonymous mutation [12]. The main sources of SNVs encompass processes such as DNA damage and replication errors, DNA mismatch repair, and defective DNA repair mechanisms [13]. Identifying neoantigens generated by SNVs can be performed using DNA sequencing (e.g., whole genome sequencing and whole exome sequencing) data. Due to the simplicity of the mutation process, which often involves altering just a single amino acid to generate neoantigens, coupled with the straightforward of their identification, research on SNVs-derived neoantigens has progressed most extensively. Their mutational characteristics make them more suitable as candidate immunotherapy targets for tumor types with higher mutation burdens, such as non-small cell lung cancer, bladder cancer and melanoma [14]. SNV-derived neoantigens have been the focus of extensive research in the field. Personalized neoantigen vaccines targeting SNV-derived neoantigens have progressed to clinical trials for melanoma treatment [15]. Similarly, SNV-derived neoantigens had emerged as viable targets for glioblastoma immunotherapy [16]. Nevertheless, the fact that these neoantigens originate from single nucleotide variations leads to a limited distinction from self-antigens, thereby may limit specificity and pose challenges in their recognition by the immune system [17].

### Insertions and deletions

In addition to SNVs, frameshift peptides (FSPs) generated by INDELs represent another significant source of mutation-derived neoantigens. FSPs are generated by insertions or deletions of base pairs within the gene sequence, and referred to as non-synonymous novel open reading frames (ORFs). Typically, FSPs encompass the amino acid sequence extending from the first new residue generated by INDEL mutation to the subsequent termination codon [18]. Furthermore, in-frame INDEL could result in the insertion or deletion of one or more amino acids without disrupting the overall reading frame. Mutant peptides generated by in-frame alterations also have the potential to serve as neoantigens [19]. In contrast to SNVs modifying a single amino acid, INDELs can cause more extensive alterations in amino acid sequences. As a result, the proteins produced after such mutations exhibit lower similarity to the original proteins and possess greater immunogenicity potential compared to SNV-derived peptides [20]. Due to their low frequency, INDEL-induced mutations typically do not elicit the same neoantigens across different patients. The only exception occurs in diseases associated with mismatch repair (MMR) deficiency. MMR deficiency leads to the accumulation of insertions and deletions, resulting in microsatellite instability (MSI). Microsatellites are sequences prone to high mutation rates, and thus MSI increases the risk of tumorigenesis. Existing studies have demonstrated that MSI-high (MSI-H) related cancers, such as colorectal cancer [21] and gastric cancer, harbor shared MSI-associated neoantigen epitopes among different patients. Consequently, INDEL-driven MSI-H tumors represent a significant source of shared antigens [22].

### Gene fusion

Gene fusion refers to that result in gene chimeras, encompassing gene fusions and gene hybrids resulting from processes such as chromosomal translocations [23]. The rearranged genome resulting from gene fusion is expressed as a chimeric protein, which also serves as a source of neoantigens. Gene fusions, particularly the frameshift fusion subtype, can induce sequence alterations and generate novel ORFs [24]. And it could generate new amino acid sequences, which possess more potential T-cell epitopes compared to SNVs and INDELs, thereby exhibiting higher immunogenicity [25]. Therefore, gene fusions have the potential to produce a greater number of neoantigens compared to SNVs and INDELs for an equivalent mutation count. However, due to the limited number of gene fusions, research progress on neoantigens based on gene fusion has not been significant. Several studies have investigated the potential applications of these neoantigens in cancer therapy. For example, Kirk et al. demonstrated that neoantigens generated by gene fusion can serve as therapeutic targets for liver cancer fibrolamellar carcinoma (FLC) [26]. Biernacki et al. proposed that the neoantigen generated by the CBFB-MYH11 gene fusion represented a potential target for immunotherapy in acute myeloid leukemia (AML) [27]. Gao et al. confirmed that the fusion genes TMPRSS2-ERG, CCDC6-RET, and FGFR3-TACC3 had the potential to become neoantigen candidates [28].

### Structural variations

Structural variations (SVs) constitute a significant category of genomic alterations. SNVs and INDELs represent short genomic variations, impacting regions of less than 50 base pairs (bp). Defined as changes involving more than 50 bp, SVs encompass a wide range of genomic modifications, including single gene insertion and rearrangements that can affect entire chromosomes [29]. Generally, SVs can be divided into balanced SV and unbalanced SV according to different generation methods. Balanced SVs denote alterations resulting from chromosomal inversions and translocations, whereas unbalanced SVs are characterized by large-scale insertions and deletions. In addition to having a significant impact on the genome, SVs have also been demonstrated to be widely distributed across various tumor types, having been observed in the development of 2,429 tumors [30]. They had the capacity to fragment a genomic region into multiple segments or fuse distinct gene regions, resulting in novel gene fragments. These fragments can be translated into peptides that hold potential as neoantigens [31]. Therefore, SVs exert a more substantial influence on the genome, and the neoantigens derived from them demonstrate higher immunogenicity [32, 33]. Wang et al. recently demonstrated that the fusion gene CLDN18-ARHGAP generated by SVs could be a promising target for immunotherapy of gastric cancer (GC) [34]. This discovery helped address a key challenge in gastric cancer, where the relatively low burden of SNVs and INDELs limits the availability of neoantigen-based therapeutic targets. Those cases indicate that neoantigens generated by SVs are an important supplement to those produced by SNVs and other mutations. Gene fusions and SVs could generate more novel ORFs compared to SNVs and INDELs, resulting in a higher abundance of neoantigens. These neoantigens are more likely to elicit robust immune responses, with frameshift fusions producing neoantigens exhibiting superior immunogenicity compared to in-frame fusions [28].

### RNA dysregulation

In addition to somatic mutations that can generate neoantigens, RNA dysregulation in tumor cells can also lead to protein variations and thereby produce neoantigens. Cancers such as meningioma, chronic lymphocytic leukemia (CLL), and glioblastoma, which exhibit low mutational burdens, often lack mutation-based neoantigens [35]. To extend the application of novel immunotherapies to these cancer types, researchers have begun exploring neoantigens derived from alternative sources [36]. In this context, RNA dysregulation has gained significant interest. RNA dysregulation plays a profound role in the expression of genomic information and is a key contributor to transcriptional diversity. RNA dysregulation, exemplified by RNA splicing and RNA editing, generates diverse transcripts and produces novel peptide sequences that can be expressed as neoantigens.

### RNA splicing

RNA splicing is a crucial step in human gene expression, enabling the conversion of pre-mRNA into mature mRNAs [37]. RNA splicing consists of constitutive splicing and alternative splicing. Constitutive splicing generally involves the removal of introns and the remaining exons to produce only one mature mRNA, while alternative splicing plays a key role in generating mRNA diversity. In general, alternative splicing generates mRNAs with differences in untranslated regions (UTRs) or coding sequences through mechanisms such as cassette exons (CE), intron retention (IR), mutually exclusive exons (MXE), and alternative 5’ or 3’ splice site usage (A5SS/A3SS) [38]. During tumorigenesis, alternative splicing can also occur due to inappropriate expression or other factors and leads to abnormal splicing events [39]. The process of mature mRNA formation can generate novel exon-exon junctions and splice sites, those sites can potentially be translated into unique antigenic peptides. These antigenic peptides exhibit tumor specificity and represent an important source of neoantigens. Kahles et al. [40] analyzed more than 8,700 samples across 32 cancer types from The Cancer Genome Atlas (TCGA) database and found that RNA splicing events in tumor samples were 20% more frequent than in normal samples, suggesting RNA splicing in tumor can generate specific RNA transcripts that are subsequently translated into tumor-specific proteins. A study [41] confirmed that mutations occurring near Alu elements in intronic regions were particularly prone to inducing aberrant splicing events, which can generate novel cancer-specific exons. A recent study also confirmed that RNA splicing can generate public neoantigens, which are present across a variety of cancers including prostate cancer, and hold the potential to serve as novel targets for cancer therapy [42]. Furthermore, Ji et al. [43] proposed that neoantigens arising from long-range alternative splicing events on circular RNAs (circRNAs) could serve as potential targets for immunotherapy in glioblastoma (GBM). In addition, circRNAs generated through back-splicing can also serve as an important source of neoantigens, which will be discussed separately in the noncanonical transcripts section.

### RNA splicing

Mutations in non-coding regions constitute 99% of cancer-associated mutations. Recent evidence has demonstrated that antigens transcribed from non-coding genomic regions and UTRs can elicit potent T cell responses [44]. Such antigens are referred to as non-canonical neoantigens and have garnered significant research interest due to their therapeutic potential. Merlotti et al. discovered that non-canonical splicing junctions linking exons and transposable elements (TEs) represented a significant source of neoantigens in non-small cell lung cancer (NSCLC) [45]. Laumont et al. suggested that neoantigens derived from non-coding regions are more suitable as targets for immune responses compared to those from coding regions. They proposed the non-canonical translation resulting in neoantigens have the potential to be shared among various cancer types [46]. Tang et al. demonstrated that circBIRC6, a circular RNA derived from back-splicing of the BIRC6 gene, is significantly expressed in gastric cancer tissues and can serve as a therapeutic target for gastric cancer [47]. Ren et al. proposed that circRAPGEF5 and circMYH9 from circRNAs can generate neoantigens with therapeutic potential for colorectal cancer (CRC) [48]. Song et al. demonstrated that circFAM53B can be presented by T cells and triggers an immune response targeting breast cancer [49]. Wang et al. predicted and validated using tumor organoids that antigenic peptides derived from circRNAs such as circTBC1D15 can serve as targets for immunotherapy [50]. Ferreira et al. proposed a workflow utilizing MS immunopeptidomics to identify circRNA-derived peptides and successfully detected 21 novel tumor-associated antigens as potential targets in melanoma and lung cancer datasets [51]. The aforementioned examples demonstrated distinct biological properties of non-canonical neoantigens.

### RNA editing

In addition to RNA splicing, RNA editing represents another significant form of RNA dysregulation. RNA editing primarily occurs in Alu sequence regions, particularly in double-stranded RNA (dsRNA) structures formed by inversely oriented Alu elements. RNA editing process can induce modifications in RNA sequences, which may subsequently lead to changes in amino acid composition. For instance, the conversion of adenosine (A) to inosine (I) editing on 3’UTR, mediated by adenosine deaminases ADAR1 and ADAR2, can lead to alterations in amino acid sequences. Therefore, the transcripts produced by it also can be recognized by T-cell receptors (TCRs) and become neoantigens. Zhang et al. [52] demonstrated that novel peptide sequences generated through RNA editing can be presented to T cells by HLA molecules. Li et al. [53] discovered that RNA editing-derived neoantigens can serve as potential targets for immunotherapy in hepatocellular carcinoma (HCC) along with somatic mutation-derived neoantigens. In addition to the extensively studied A-to-I editing, cytosine (C) to uracil (U) editing is another form of RNA editing. This modification process is catalyzed by the APOBEC family of editing enzymes [54]. C-to-U editing has the potential to produce neoantigens through modifications in RNA sequences, which are subsequently translated into altered proteins [55].

While RNA-derived neoantigens significantly broadened the landscape of potential tumor targets, it was crucial to acknowledge their potential limitations, particularly concerning their stability as immune targets. Unlike neoantigens arising from somatic DNA mutations, which were coded into the genome of tumor cells, RNA-based epitopes could be more dynamic. The expression of aberrant transcripts or dysregulated isoforms might be lost under immune pressure through several mechanisms [56]. For instance, tumors could evade T-cell recognition via altering the expression of splicing factors or undergoing broader transcriptional changes that downregulate or eliminate the transcript encoding the target neoantigen [42]. This plasticity meant that RNA-derived neoantigens might be less stable, potentially allowing tumor cells to develop resistance and escape immune control [57]. Moreover, RNA dysregulation often involves changes in the percentage of exon or intron inclusion. This could lead to clonal consistency [58].

### Protein modifications

Beyond investigating neoantigen origins through genomic and transcriptomic approaches, proteomics has also identified mutated peptides as a focal point of research. Advances in mass spectrometry technology now enable the direct analysis of peptides bound to major histocompatibility complexes (MHC) molecules, revealing that post-translational spliced peptides and post-translational modifications (PTMs) produce self-generated neoantigens. A form of PTM, MUC1 aberrantly glycosylated, was a recognized tumor specific antigen uniquely associated with epithelial cell tumors [59]. Phosphorylated epitope from cell division cycle 25B (CDC25B) was overexpressed in pancreatic cancer (targeting ENO1), while citrullinated epitopes from ENO1 and metallopeptidase 21 (MMP21) were specifically upregulated in ovarian cancer and melanoma, respectively [60].

### Post-translational spliced peptides

The study of alternative splicing at the proteomic level originated with the discovery of spliced antigens, which are generated through the fusion of two nonlinearly encoded peptide segments. This generation process is facilitated by proteasomes and takes place during proteasomal cleavage. Proteasomal cleavage is a necessary step for generating complete proteins in the cytoplasm. However, proteasomes frequently produce spliced peptides that diverge from the original protein sequences through the excision and recombination of peptide segments during proteasomal processing [61]. Proteasome-mediated peptide cleavage results in the formation of both cis-spliced and trans-spliced peptides. Several recent studies [62, 63] have demonstrated that the aforementioned spliced peptides are capable of being recognized by CD8 + T cells, thereby confirming their immunogenicity and establishing them as a novel source of neoantigens. Neoantigens generated through peptide splicing have been demonstrated to elicit immune responses targeting melanoma [64]. Fidanza et al. discovered that targeting proteasome-catalyzed splicing enhanced the therapeutic success rate in glioblastoma [65]. However, due to the low occurrence frequency of spliced peptides, further mass spectrometry data are required to advance research on neoantigens driven by spliced peptides [66].

### Post-translational spliced peptides

Numerous studies have demonstrated that peptides bearing PTMs can elicit immune responses via mass spectrometry analysis [67]. Common PTMs include phosphorylation, methylation, and acetylation [68]. PTMs affect protein function in various ways, such as by modulating protein structure and protein-ligand interactions. Similarly, PTMs can alter protein binding conformations, enabling them to bind with MHC molecules and generate neoantigens. Specifically, PTMs can further modify amino acid structures, resulting in novel TCR-binding peptides that interact with T cells in a manner distinct from the original peptides, potentially eliciting autoimmune responses. Using the developed platform PROMISE, Kacen et al. found a significant increase amount of phosphorylated peptides bound to HLA in breast cancer and demonstrated that PTM can generate novel HLA I-binding motifs [69]. Such modified antigens can provide new therapeutic opportunities for cancer immunotherapy. Brentville et al. demonstrated that citrullinated peptides can elicit CD4 T cell responses, showing potential as targets for cancer immunotherapy [70]. Zhai et al. demonstrated that neoantigens produced through cysteine carboxymethylation modifications had the potential to act as therapeutic targets for the immune disease ankylosing spondylitis (AS) [71]. Current limitations in protein characterization technologies have restricted the identification of PTM-derived neoantigens. Advances in MS platforms have the potential to expand the range of detectable PTM neoantigens, thereby enriching the neoantigen landscape (See Fig. 1).

### A representative workflow for neoantigen prediction

**Fig. 2:**
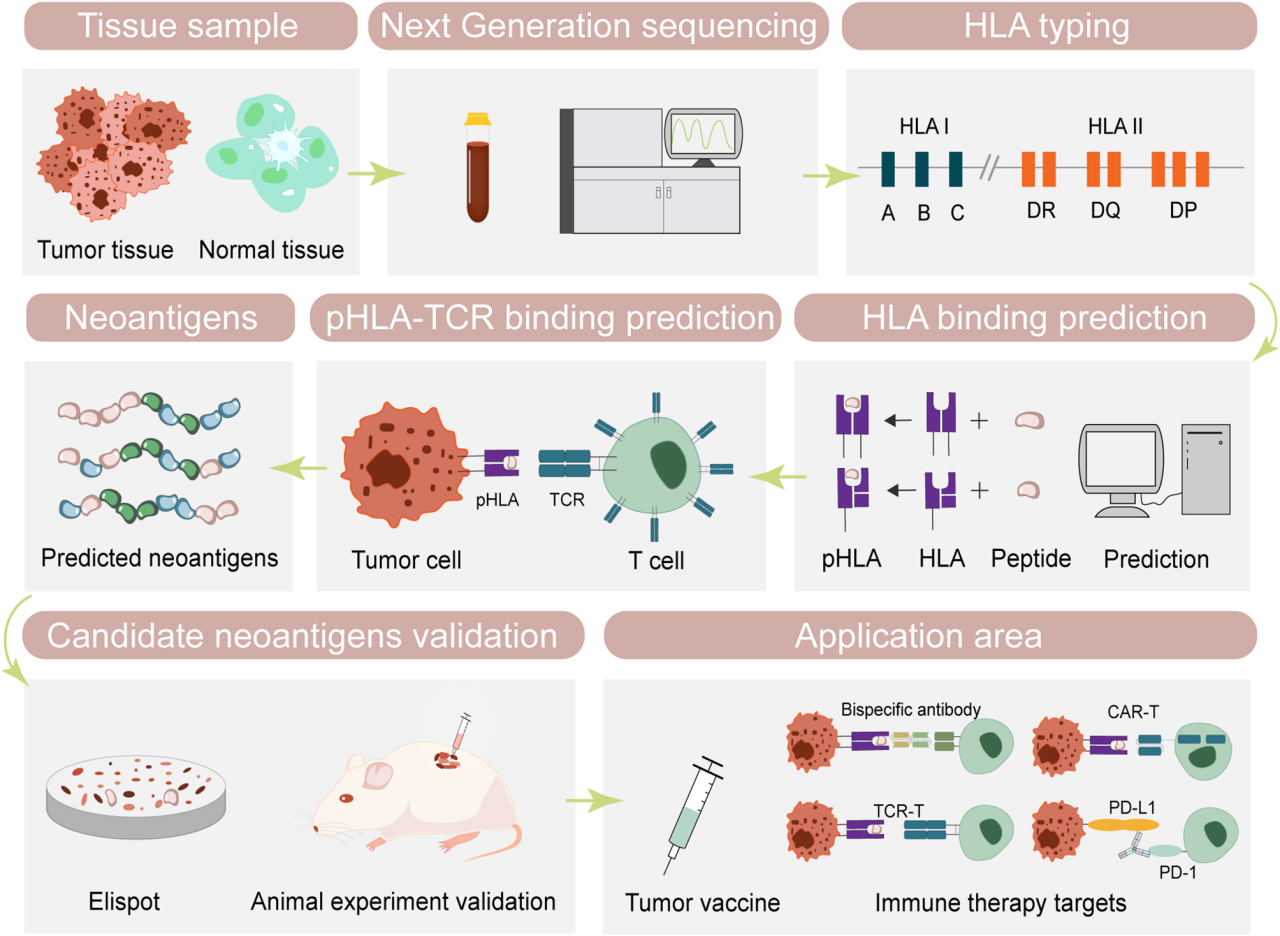
A representative discovery of neoantigen prediction. Collecting tumor tissues and normal tissues such as para-carcinomatous tissues or peripheral blood mononuclear cells (PBMC) for sequencing. Subsequently, HLA typing is conducted to ascertain accurate HLA subtypes. Further, to generate candidate neoantigens employing Peptide-HLA-TCR binding prediction algorithms. Finally, the immunogenicity of candidate neoantigens is validated through enzyme-linked ImmunoSpot (ELISpot) assays or mouse animal experiments. The validated neoantigens are subsequently utilized for immunotherapy

With the rapid advancements in sequencing technologies and artificial intelligence algorithms, existing methods have significantly enhanced the prediction of neoantigens from various sources. Neoantigens will elicit immune responses when their peptides are bound to MHC molecules and presented on the surface of antigen-presenting cells for recognition by T cells. Therefore, these two processes are critical components in neoantigen prediction, forming the basis of the accuracy of the results. In this section, we will focus on the methodologies in neoantigen prediction workflow. The schematic overview of the discovery is illustrated in Fig. 2.

### HLA typing

**Fig. 3:**
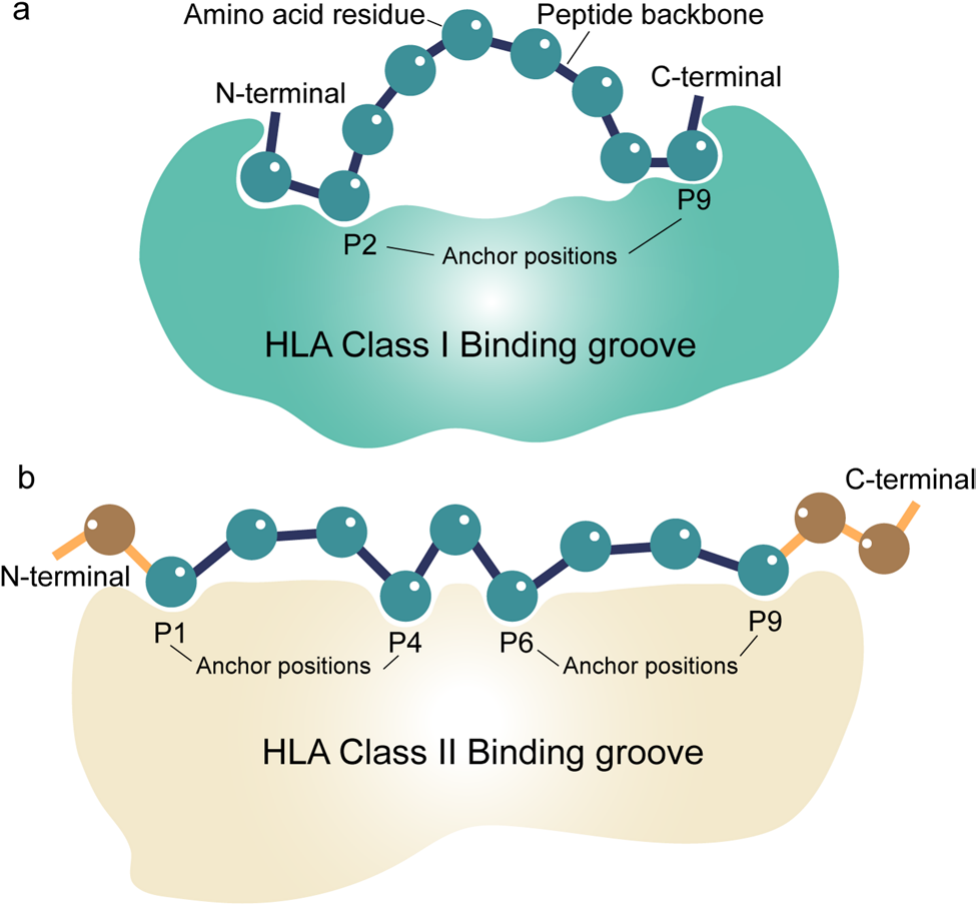
Peptide-binding grooves of HLA class I and II. (**a**) HLA class I binding grooves are suitable for peptides 8–10 amino acids long. (**b**) HLA class II binding grooves are suitable for peptides 13–18 amino acids long. In addition, HLA class I has a closed groove topology while HLA class II has an open groove topology. For the HLA Class I binding groove, P2 and P9 are common anchor residues. The core 9-mer is the key binding region for the HLA Class II binding groove

HLA typing constitutes the first step in neoantigen prediction. The antigen-binding groove of MHC molecules forms stable peptide-MHC (pMHC) complexes upon successful engagement with neoantigens. Subsequently, this pMHC complex can activate cognate T cells, initiating specific immune responses. In humans, MHC molecules are referred to as human leukocyte antigens (HLAs), with the HLA-I and HLA-II subtypes playing predominant roles in neoantigen presentation [72]. For CD8^+^ T cells, priming occurs via antigen presentation on HLA-I molecules by professional APCs, followed by direct recognition of tumor cells presenting the same neoantigen via HLA-I. For CD4^+^ T cells, neoantigen peptides must be presented on HLA-II molecules, again typically by professional APCs [73]. Furthermore, HLA-I is expressed on all nucleated cells and can further be classified into HLA-A, HLA-B, and HLA-C. HLA-I molecules typically bind peptides of 8–10 amino acids in length, which represents the optimal range due to the structural constraints of their closed-ended peptide-binding groove. Position 2 (P2) and position 9 (P9) within the HLA class I peptide-binding groove are critical anchor residues for binding neoantigen peptides [74]. In addition, longer peptides can also be tolerated through bulging conformations, whereas shorter peptides exhibit suboptimal binding affinity [75–77]. HLA class II molecules are classified into HLA-DR, HLA-DQ, and HLA-DP [78]. HLA-II molecules typically bind peptides of 13–18 amino acids in length, with a central core of 9 residues forming stable interactions within the open-ended peptide-binding groove [79]. HLA-II molecules are primarily expressed by APCs. However, IFNγ and other stimuli can drive HLA-II expression in non-APCs. This tumor-intrinsic HLA-II expression may facilitate direct antigen presentation to CD4^+^ T cells, broadening the functional roles of HLA-II beyond classical immune cell subsets [80]. As demonstrated in Fig. 3, HLA class I and class II molecules exhibit differences in antigen-binding groove architectures and peptide interaction modes.

In addition to its numerous subtypes, HLA exhibits high inter-individual variability. Consequently, the HLA alleles of each patient determine the specific neoantigens they can present, and precise HLA typing can enhance the accuracy of neoantigen prediction [79]. Selecting candidate neoantigens based on patient-specific HLA typing is essential for activating T-cell responses. ArcasHLA [81] used the RNA quantifier method Kallisto for pseudo-alignment feature to perform HLA gene alignment. HISAT-genotype [82] employed a graph-based Ferragina-Manzini index to align genetic sequences, enabling HLA typing. HLA-HD [83] improved the accuracy of HLA typing by weighting exons corresponding to the G-DOMAIN and extending the analysis to other exons. HLAProfiler [84] was a k-mer profile-based tool for accurate and comprehensive HLA typing from RNA-seq data. OptiType [85] was specifically designed for HLA class I typing and demonstrated outstanding performance. ArcasHLA, HISAT-genotype, HLA-HD and HLAProfiler were capable of performing excellent HLA typing for both HLA class I and HLA class II data. Furthermore, it is essential to exclude the influence of HLA mutations that lead to a loss of antigen presentation function during the prediction pipeline.

### HLA binding prediction

In the process of neoantigen presentation, T cells can only recognize neoantigens presented by HLA molecules. Consequently, predicting the binding affinity between HLA molecules and tumor neoantigens constitutes the initial step in neoantigen prediction. NetMHCpan [86] used public datasets for training and employed the NNAlign MA framework for data processing. MHCflurry [87, 88] employed the same feedforward neural networks (FFNN) algorithm as NetMHCpan. MHCflurry, NetMHCpan, NetMHC [89] and MixMHCpred [90, 91] achieved excellent predictive performance through continuous iterative updates. The binding performance evaluation revealed that MixMHCpred excels in predicting the interactions between HLA-I and peptides [92]. Inspired by the success of Convolutional Neural Networks (CNNs) in other fields, methods such as ACME [93] and Seq2Neo [94] have introduced CNNs into the realm of peptide-HLA (pHLA) binding prediction. The framework hierarchically integrates multi-scale features extracted from different CNN layers to improve the representation of peptide-MHC binding. CapsNet-MHC [95] identifies the limitations of feature fusion models in capturing the intricate relationships between peptide and MHC sequences, and innovatively integrates the capsule neural network with CNN to model the ordered relations within the extracted learned features.

HLApollo [96], HLAB [97] and TransPHLA [98] incorporated the Transformer model within its peptide-MHC-I prediction framework, leveraging the capability of Transformers to learn features for further refining the characteristics of protein sequences. TripHLApan [99] had recently incorporated a triple encoding matrix, a BiGRU + Attention model, and a transfer learning strategy to predict the binding interactions between HLA molecules and peptides. TripHLApan employed parallel encoding of multiple amino acid features to capture more comprehensive global information, while simultaneously applying residue-level weighting to better reflect the various importance of different amino acid residues positions, ultimately enhancing prediction accuracy. TransHLA [100] achieved leading prediction results by leveraging large language models for sequence embedding, while also incorporating both Transformer and Residue CNN architectures. MUNIS [101] was one of the latest HLA-I-peptide binding prediction methods, employing a bimodal architecture that integrates a protein language model and a peptide-flanks encoder and capitalizing on the superior encoding capabilities of large language models. Meanwhile, it shows excellent performance in predicting new CD8^+^ T cell epitopes. Utilizing the aforementioned methods to predict the binding affinity of mutated antigen peptides to HLA, the peptides exhibiting the highest affinity are identified and input into the subsequent prediction pipeline. Additionally, many methods use HLA eluted ligand (EL) datasets identified by mass spectrometry for prediction. ConvNeXt-MHC [102], BigMHC [103], and NetMHCpan are representative algorithms in this domain.

The prediction of peptides that bind to HLA class II molecules was critical for identifying HLA class II restricted neoantigens. Due to the lack of HLA-DQ-specific antibodies, HLA class II binding prediction studies had predominantly focused on HLA-DR typing, which had hindered the comprehensive development of HLA class II research and resulted in its predictive accuracy lagging behind that of HLA class I [104]. NetMHCIIpan, one of the earliest specific HLA class II binding prediction tools, had evolved into its current version NetMHCIIpan-4.3, which significantly enhances predictive performance by incorporating the updated NNAlign_MA architecture and integrating the latest immunopeptidomic training datasets [72]. MixMHC2pred employed two neural network blocks: the first predicts binding specificity (PWM) from MHC-II allele sequences, and the second used the PWM and peptide sequences to predict peptide presentation [105]. TLimmuno2 developed a transfer learning-based LSTM framework for accurate prediction of HLA class II presented epitopes [106]. Graph-pMHC was a novel MHC class II peptide presentation model that leveraged AlphaFold2-multimer (AF2)-derived structural adjacency matrices and an alignment-based binding core localization strategy [107]. NetMHCIIpan remained the gold standard for MHC-II binding prediction while MixMHC2pred enhanced its framework through integration of MS-eluted ligand datasets. TLimmuno2 extended predictive capabilities to immunogenicity assessment and Graph-pMHC introduced structure-aware modeling as a transformative paradigm. These tools illustrated the evolution of the field.

### pHLA-TCR binding prediction

**Fig. 4:**
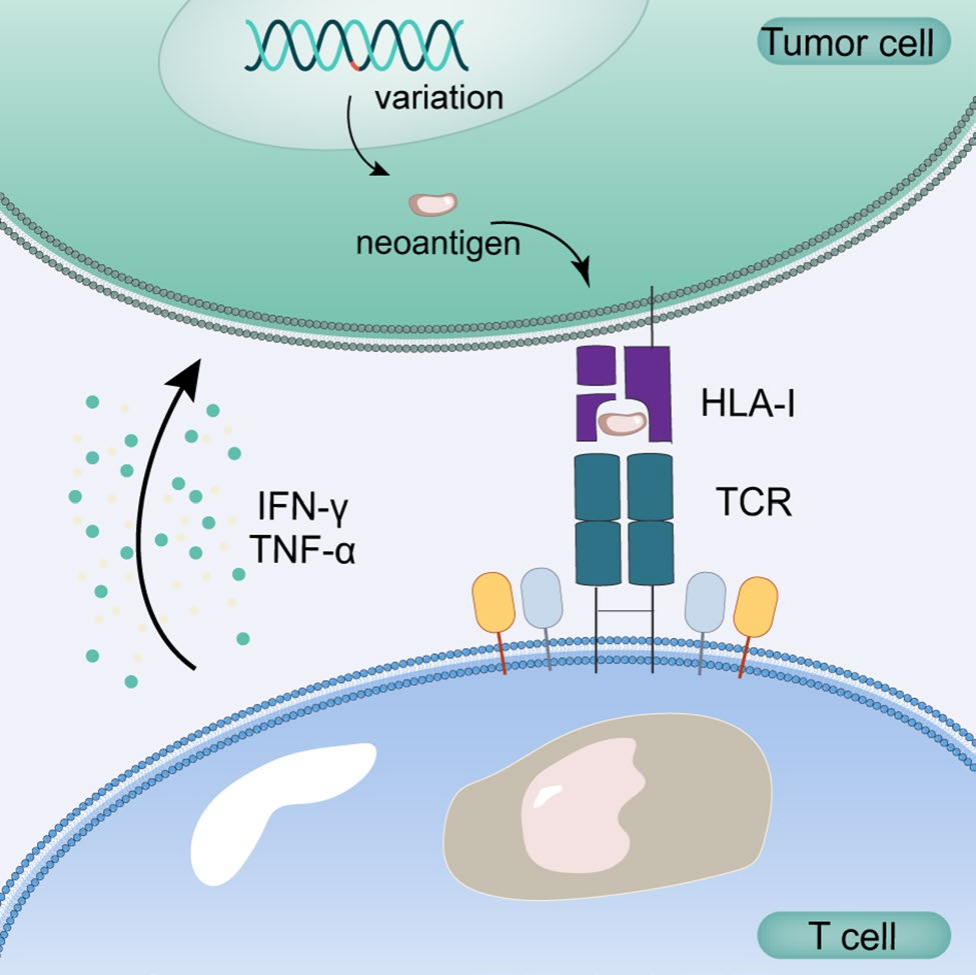
The binding between neoantigens, HLA and TCR

The recognition of MHC-presented peptides by T cells constitutes a key step in neoantigen prediction. As shown in Fig. 4, the interaction between HLA-peptide complexes and TCRs provides a predictive reference. Models for peptide-specific TCR binding prediction predominantly utilized the complementarity-determining region 3 (CDR3) sequence of the TCR β chain as TCR sequence [108]. A variety of machine learning models had been proposed for peptide-TCR binding prediction, including DeepCAT [109], DeepTCR [110], ATM-TCR [111], TITAN [112], DLpTCR [113], CATCR [114], NetTCR-2.0 [115], P-TEAM [116], TCRGP [117], and TCRdist [118]. Although these models employed diverse techniques like CNN and attention mechanisms, they were often limited by challenges in handling non-linear feature relationships and ensuring model interpretability.

In order to better quantify the direct binding between TCR and peptide, numerous models have proposed their effective strategies. GTE [119] employed a heterogeneous graph neural network (GNN) based on inductive learning as its core framework, modeling the binding interactions between TCRs and neoantigen epitopes as an edge classification task. For the purpose of effectively utilizing the topology between TCR and epitope, GTE uses graph learning methods to model the nonlinear relationship between TCR and epitope. HeteroTCR [120] also adopted a Heterogeneous Graph Neural Network and further enhanced the capture of high-order neighborhood interaction information. GGNpTCR [121] and DiffRBM [122] employed a Self-Generating Graph Attention Network (SGGAN) and a differential learning strategy to better capture the spatial structural information of amino acid sequences. TEIM [123] addressed the limitations in the residue-level field by designing a novel interaction extractor, which accomplishes the residue-level binding prediction task. DeepAIR [124] and TCRpcDist [125] successfully enhanced their predictive accuracy by integrating 3D structural information of CDR3 into their models.

Existing prediction methods often relied on training with known sequences and thus performed poorly when faced with unknown sequences. Many methods had been improved to address this shortcoming. Panpep [126] pioneered the integration of meta-learning and neural Turing machines. Panpep transformed peptide-TCR recognition into a set of tasks in a meta-learning framework, improving the generalization ability of novel peptides. EPACT [127] employed supervised contrastive learning for epitope-pMHC anchors to maintain epitope specificity, complemented by transfer learning to improve model adaptability. TULIP [128] innovatively integrated transformer architecture and natural language processing (NLP) models to improve the generalization capability of the prediction model for unknown peptides. SC-AIR-BERT [129] introduced the BERT model to improve prediction accuracy. tcrBLOSUM [130] introduced two novel amino acid matrices, tcrBLOSUMa and tcrBLOSUMb, designed for the CDR3α and CDR3β TCR chains respectively, enabling the retention of more comprehensive TCR information. Methods such as TEINet [131], MixTCRPred [132], MITNet [133] and T-FINDER [134] had also achieved outstanding performance by integrating Transformer architectures, CNNs, fully connected neural networks, and other techniques.

**Fig. 5:**
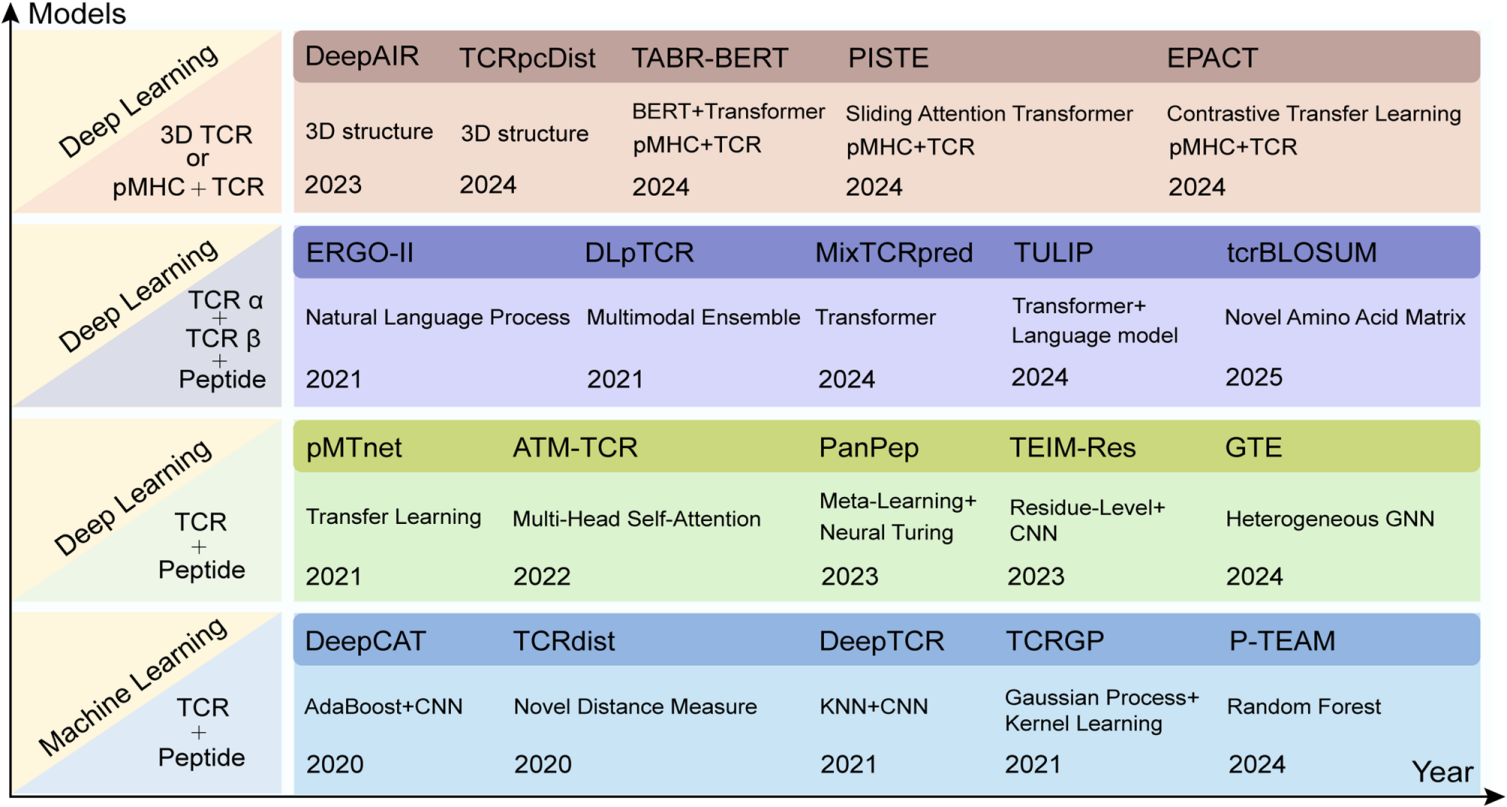
The evolution of technology for TCR and peptide binding prediction algorithms

The aforementioned prediction methods exclusively incorporate peptide and TCR information, neglecting MHC data, thereby diminishing their capability to predict unknown antigen epitopes. ERGO-II [135] pioneered the expansion of model inputs by integrating the CDR3 sequences from both the TCR α and β chains, as well as peptide and MHC sequences, into the training process to mitigate experimental bias. pMTnet [136] employed transfer learning to simultaneously model CDR3 sequences, antigen sequences, and MHC-I inputs. TABR-BERT [137] integrated CDR3β sequences, epitopes, and MHC information into the BERT model to transfer learning. PISTE [138] developed a sliding-attention module that concurrently quantifies the interactions between HLA, antigens and TCRs. UnifyImmun innovatively designed a two-phase strategy, enabling the encoder of the unified cross-attention transformer model to extract more binding information of peptide-HLA and peptide-TCR [139]. UniPMT had recently integrated three prediction tasks: peptides-MHC-TCR, peptides-MHC, and peptides-TCR, to incorporate more binding information and further improve the accuracy [140]. In Fig. 5, we presented a comprehensive overview of representative prediction algorithms currently, along with their core technologies.

Current prediction models are prone to overfitting the training dataset, which constrains their generalization capabilities. This represents a critical area for enhancement in future model iterations. Moreover, existing models exhibit limited computational efficiency when handling large datasets and demand significant computational resources. Future efforts should focus on optimizing computational efficiency while maintaining high prediction accuracy.

### Performance comparison of prediction tools

To enable effective comparison among diverse predictive tools, equitable evaluation using a standardized dataset was essential for achieving an intuitive assessment of their performance. Wu et al. developed a large-scale benchmark dataset comprising 44 HLA-I alleles and over 290,000 peptides to systematically assess the performance of HLA class I peptide-binding prediction tools [141]. This work evaluated the performance of seventeen commonly used peptide-HLA binding prediction methods, including STMHCpan, NetMHCpan4.1_EL, MHCflurry2.0_BA, CapsNet-MHC, TransPHLA, NetMHCpan4.1_BA and DeepHLApan, across metrics such as Precision, F1-score, Accuracy, and MCC. The analysis revealed that STMHCpan [142], MHCflurry2.0_PS [87] and BigMHC [127] achieved the highest scores across distinct evaluation metrics. STMHCpan, MHCflurry2.0_PS and BigMHC achieved their success by employing advanced deep learning architectures to more accurately model the complexities of neoantigen processing and presentation.

For TCR-pHLA binding prediction, the recent PISTE [138] method compared seven prediction tools: TEIM, ImRex, Panpep, NetTCR, EGRO-AE, EGRO-LSTM and TEINet, across three metrics: area under the receiver operating characteristic curve (AUROC), area under the precision-recall curve (AUPR) and positive predictive value at top-n (PPVn). To ensure robustness against methodological variability, three distinct negative sample construction methods: random sequence shuffling, unified epitope modeling, and reference TCR alignment were integrated into the dataset generation process, encompassing 607 neoantigens presented by 65 HLA class I molecules and 29,687 TCR sequences. Excluding PISTE, TEIM [123] and ImRex [108] were the other top-performing methods across all metrics. Subsequent TCR-pHLA binding prediction tools were recommended to focus on benchmarking against these validated methods.

### Integrated neoantigen prediction algorithms

Beyond predicting peptide-MHC binding affinity, which was a necessary first step, modern neoantigen prediction workflows incorporate several additional features to prioritize candidates with the highest therapeutic potential. These criteria were essential for estimating the likelihood that a neoantigen will be successfully processed, presented, and recognized by T cells in vivo. Neoantigen immunogenicity was determined by factors including the RNA-seq quantified expression level of the mutated gene, clonality, proteasomal processing, TAP-mediated transport efficiency, agretopicity - the ratio of mutant binding affinity to wild-type binding affinity [143]. These influencing factors could provide a more precise measure of binding stability than affinity alone. Neopepsee categorized potential immunogenic features into three classes based on genomic and proteomic data: MHC-I binding and presentation, amino acid characteristics and complex scores [144]. The ICERFIRE ensembled random forest model, constructed through comprehensive analysis of peptide features including BLOSUM mutation scores and antigen expression levels, demonstrated predictive capability for the immunogenicity of cancer neo-epitopes [145]. Predicting neoepitope immunogenicity model IMPROVE was rigorously validated across large-scale, multi-cohort T-cell response datasets to identify key determinants, such as the presence of hydrophobic and aromatic residues in the peptide binding core, peptide-HLA binding affinity and sequence context [146]. By annotating and analyzing epitope residues, PRIME could decouple HLA-I binding from molecular TCR recognition propensity, thereby enabling a more accurate prediction of peptide immunogenicity [147]. Furthermore, pipelines such as Neo-intline [148] enabled the prediction of the likelihood of each mutation-containing peptide (MCP) being presented and subsequently recognized by T cells, starting from mutations identified via whole-genome sequencing (WGS). These pipelines had been validated for immunogenicity through both in vitro and in vivo experiments. Such successful workflows could serve as established benchmark pipelines for neoantigen prediction.

### Neoantigen-based immunotherapy

Existing tumor treatments, such as chemotherapy and radiotherapy, generally treat tumor by disrupting the normal cellular activities of tumor cells. However, these treatments lack precision in targeting and eliminating tumor cells, and lead to harm to numerous healthy cells in the process. In this context, emerging immunotherapy has become promising research for tumor treatment. Currently, immunotherapies utilizing neoantigens as targets primarily encompass cancer vaccines, adoptive cell therapy, and bispecific antibodies.

### Cancer vaccine

Despite decades of conceptual validation, cancer vaccines have failed to convert into applicable research examples due to the limited immunogenicity of vaccine targets. The discovery of tumor antigens, especially neoantigens, has brought hope for the advancement of cancer vaccines. Neoantigen-based cancer vaccines include peptide vaccines, nucleic acid (DNA/mRNA) vaccines and dendritic cell (DC)-based vaccines, among others. A recent study demonstrated that neoantigen peptide vaccines improved overall survival in GBM patients undergoing postoperative treatment [149]. And Braun et al. revealed that personalized cancer peptide vaccines were capable of eliciting sustained immune memory in patients with renal cell carcinoma, thereby diminishing the likelihood of cancer recurrence [150]. Clinical studies on neoantigen DNA vaccines have shown that 87.5% of triple-negative breast cancer patients achieved a 3-year recurrence-free survival [151].

**Fig. 6:**
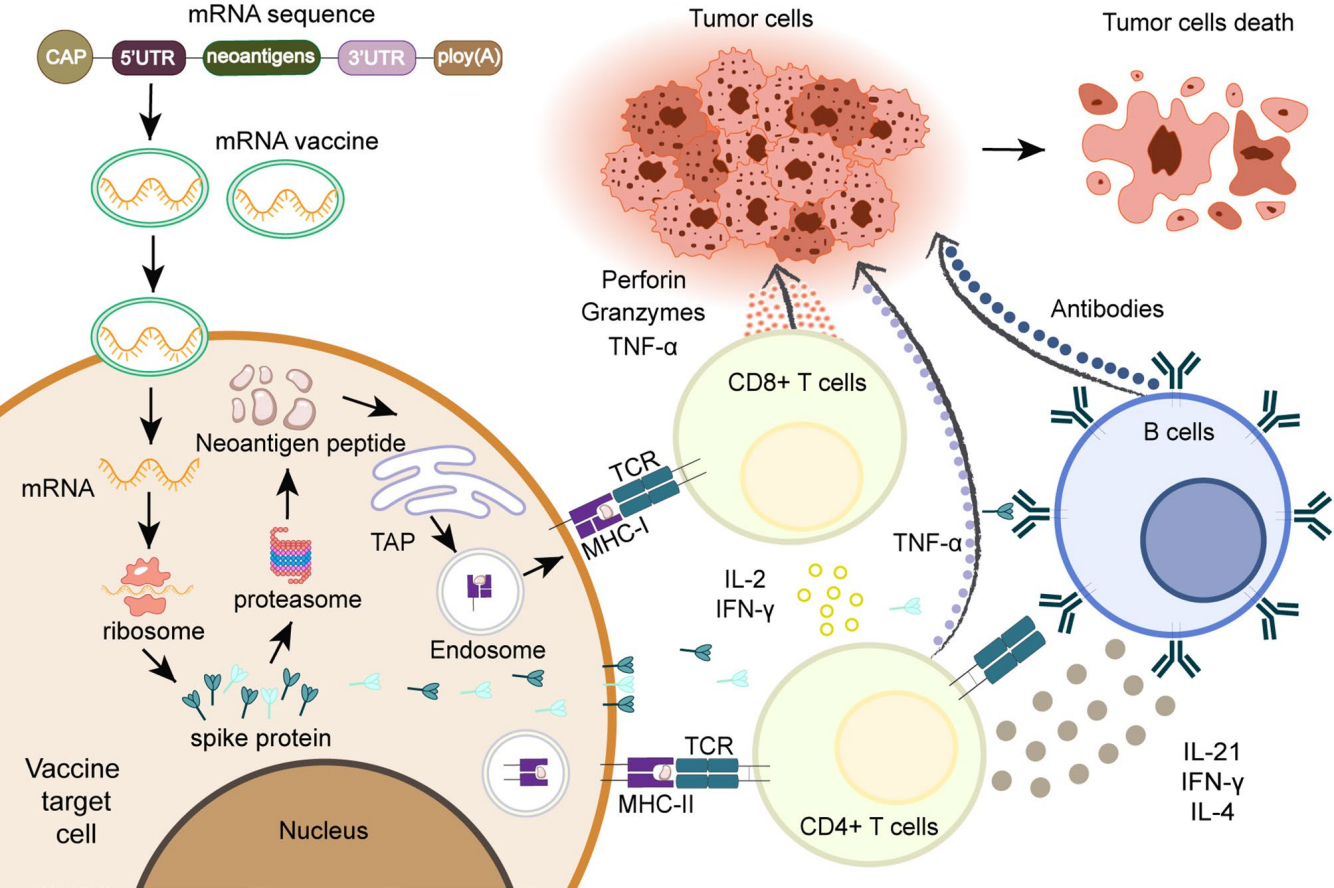
The process by which mRNA neoantigen cancer vaccines exert immune response. Firstly, the mRNA sequence encoding neoantigens is prepared into an mRNA vaccine. After activating APC, the vaccine is gradually translated to produce the neoantigen protein, which triggers an immune response against tumor cells by T cells

Owing to their success in COVID-19 vaccines, mRNA vaccines have emerged as a focal point in vaccine development. Their advantages, such as rapid production, robust thermal stability, and effectiveness at low doses, have positioned them as a promising option for cancer immunotherapy. Moderna and other partner companies had developed the neoantigen mRNA vaccine mRNA-4157, which has advanced to Phase III clinical trials. Phase 2b clinical data reveal that mRNA-4157, when combined with PD-1 antibody therapy for melanoma, reduces the risk of distant metastasis or death by 65% [152]. Another vaccine company, BioNTech, had reported that its mRNA vaccine Cevumeran (BNT122) for pancreatic cancer treatment can alleviate patient conditions. The latest clinical results show that Cevumeran could successfully elicit immune responses in CD4^+^ or CD8^+^ cells in 71% of experimental patients [153]. In addition, Cevumeran had also shown significant potential in the treatment of pancreatic cancer, and the T cells induced by it can effectively infiltrate recurrent pancreatic cancer [154]. Most of the existing mRNA cancer vaccines choose to combine with drugs such as immune checkpoint inhibitors to maintain the immune memory of T cells. Figure 6 illustrates the efficacy process of mRNA tumor vaccines targeting neoantigens.

These vaccine platforms exhibit distinct immunological profiles, with nucleic acid-based vaccines (DNA/RNA) demonstrating superior immunogenicity compared to peptide vaccines. Peptide vaccines bypass the need for electroporation-assisted delivery required by DNA vaccines or lipid nanoparticle encapsulation essential for RNA vaccines, and can be delivered directly to patients. Furthermore, mRNA vaccines offer unique advantages in rapid design and eliminate risks of genomic integration. Similarly, each vaccine platform presents its own unique technical challenges. For instance, mRNA vaccines require the development of more efficient delivery systems, DNA vaccines necessitate the design of improved electroporation devices, and peptide vaccines must address strategies to enhance immunogenicity. To overcome these limitations, a combination of different vaccines could be considered, leveraging the respective strengths of each platform to achieve optimal results.

### Adoptive cell therapy

In vitro genetic engineering enables the reprogramming of immune cells to express tumor-specific TCRs or chimeric antigen receptors (CARs), which can be utilized in ACT for cancer immunotherapy [155]. Tumor-infiltrating lymphocyte (TIL) therapy, T cell receptors-engineered T cell (TCR-T) therapy, and chimeric antigen receptor-engineered T cell (CAR-T) therapy are the three primary modalities of ACT. CAR-T therapy has been successful in the treatment of leukemia [156]. Compared with CAR-T therapy, TCR-T therapy has the potential to target a broader range of antigens. Additionally, TCR-T is more effective in attacking solid tumors in comparison to CAR-T. Nevertheless, a significant challenge remains in identifying specific targets for TCR-T therapy and CAR-T therapy. The lack of adequately specific targets may lead to immune-related adverse events in immunotherapy, such as tumor extravasation toxicity (OTOT), which are driven by off-target specificity [157]. This phenomenon occurs when immune responses mistakenly target normal tissues instead of tumor cells. By selecting neoantigens as targets, this approach enables more precise cancer cell elimination and enhances the efficacy of immune responses. A study had demonstrated that neoantigens resulting from the tryptophan deletion-induced W > F variant can serve as promising targets for TCR-T therapy [158]. Bei et al. discovered and verified the efficacy of TCR-T therapy targeting the SYT-SSX fusion neoantigen in the treatment of synovial sarcoma [159]. The current challenges preventing the widespread use of ACT therapies, such as TCR-T therapy, are the selection of neoantigen targets and the large-scale production of specific T cells.

### Bispecific antibodies

Bispecific antibodies (bsAbs) are antibodies that can simultaneously recognize and bind to two different cancer cell epitopes. In cancer therapy, it can bridge effector cells and cancer cells, facilitating the targeted killing of tumor cells. At present, a number of bsAbs therapeutic drugs have received approval for clinical application globally, and these agents are capable of inhibiting the proliferation of tumor cells [160]. Nevertheless, during the experimental phase of bsAbs drug, phenomena such as off-target binding and non-specific interactions with extraneous proteins may occur. These occurrences can result in aberrant drug release and unintended side effects, while also presenting significant challenges for the validation of therapeutic efficacy and the scalability of mass production. Following neoantigen binding, bsAbs therapy can target neoantigens on cancer cells at one end while targeting T cells at the other end to initiate immune responses. Hong et al. chose the neoantigen NPM1/HLA0201 complex as the target for bsAbs and achieved success in the immunotherapy of AML patients [161]. Hsiue et al. [162] designed and validated a bispecific antibody-based tumor immunotherapy targeting a neoantigen encoded by the TP53 gene. Experimental results demonstrated that the bispecific antibody effectively activates T cells even with low pHLA complex expression, achieving superior specificity compared to conventional tumor antigen targeting approaches.

## Challenges and opportunities for neoantigen discovery

### Multi-dimensional characterization of neoantigen

Neoantigens have a wide range of sources, with continuous identification of novel sources driven by technological advancement. Progress in next-generation sequencing (NGS) technologies has significantly expanded the availability of genomic data. The accumulation of an increased number of sequencing and clinical data can significantly promote further research on the prediction of neoantigens derived from different sources. Herein, we collected publicly accessible datasets encompassing whole exome sequencing (WES) data, RNA sequencing (RNA-seq) data, peptide data, and experimentally validated neoantigens.

### Neoantigen database

Among these databases, Immune Epitope Database (IEDB) [163] is the largest antigen epitope database and also provides HLA typing for millions of peptides. The Cancer Epitope Database and Analysis Resource (CEDAR) [164] specially collects antigen epitopes related to cancer on the basis of IEDB. FusionNeoAntigen [33] collects and analyzes human cancer fusion gene events, compiling the current fusion-specific neoantigens. More detailed description of these databases and database links are shown in Table 1.

**Table 1:** The content brief and link of the main existing neoantigen databases

Database	Data type	Year	URL
IEDB [163]	HLA \ Epitopes \ TCR	2018	https://www.iedb.org/
CEDAR [164]	HLA \ Epitopes \ Neoantigens	2023	https://cedar.iedb.org/
TESLA [143]	Epitopes \ Neoantigens	2020	https://www.synapse.org/Synapse: syn21048999/wiki/607,073
NEPdb [165]	HLA \ Epitopes \ Neoantigens	2021	http://nep.whu.edu.cn/
ITSNdb [166]	HLA \ Epitopes \ Neoantigens	2023	https://github.com/elmerfer/ITSNdb﻿
TANTIGEN [167]	HLA \ Epitopes \ Neoantigens	2021	https://projects.met-hilab.org/tadb
CAD [168]	HLA \ Mutant Genes \ Neoantigens	2022	http://cad.bio-it.cn/
IEAtlas [169]	HLA \ Epitopes \ Neoantigens	2023	http://bio-bigdata.hrbmu.edu.cn/IEAtlas
TSNAdb [170]	HLA \ Epitopes \ Neoantigens	2018	http://biopharm.zju.edu.cn/tsnadb
GNIFdb [171]	HLA \ Epitopes \ Neoantigens	2022	http://www.oncoimmunobank.cn/index.php
NeoPeptide [172]	HLA \ Epitopes \ Neoantigens	2019	https://github.com/lyotvincent/NeoPeptide
Neodb [173]	HLA \ Epitopes \ Neoantigens	2023	https://liuxslab.com/Neodb/
dbPepNeo [174]	HLA \ Epitopes \ Neoantigens	2020	https://www.biostatistics.online/dbPepNeo/
FusionNeoAntigen [33]	Neoantigens derived from gene fusion	2024	https://compbio.uth.edu/FusionNeoAntigen

**TCR database**.

TCR-pHLA binding prediction represents a key step in the neoantigen prediction workflow, making TCR databases equally critical for accurate neoantigen identification. TCR is composed of two distinct polypeptide chains, the α chain and the β chain. The CDR3 region in the variable (V) regions of both chains primarily responsible for antigen interaction [175]. Therefore, CDR3 sequences is commonly utilized as input for TCR-pHLA prediction models. Among current TCR-peptide binding prediction algorithms, a limited number of approaches consider both the α chain and the β chain as a unified CDR3 sequence, incorporating them as simultaneous inputs. In contrast, the majority of earlier algorithms use only a single chain as input. In TCR databases, some include the α chain and the β chain dual-chain sequences, while others only contain single-chain information. Numerous researchers have developed various TCR sequence databases by compiling published CDR3 sequences, along with associated antigen specificity and disease-related information. NeoTCR [176] complemented existing TCR sequence databases by adding information on neoantigens, corresponding neoepitopes, and HLA alleles. TCR 3D [177] had updated the engagement of TCR-peptide-MHC structures, including parameters such as the crossing angle and incident angle. In the Table 2, we provided a summary of the most recent TCR databases.

**Table 2:** The introduction of the main existing TCR sequence databases

Database Name	Constituent	Year	URL
McPAS-TCR [178]	α chain and β chain	2017	https://friedmanlab.weizmann.ac.il/McPAS-TCR/
ImmuneCODE [179]	β chain	2020	https://clients.adaptivebiotech.com/pub/covid-2020
VDJdb [180]	α chain and β chain	2020	https://vdjdb.cdr3.net
TCRdb [181]	β chain	2021	http://bioinfo.life.hust.edu.cn/TCRdb/
NeoTCR [176]	α chain and β chain	2024	http://www.neotcrdb.com/
huARdb [182]	α chain and β chain	2022	https://huarc.net/database
STCRDab [183]	α chain and β chain	2018	http://opig.stats.ox.ac.uk/webapps/stcrdab
TCR3d 2.0 [177]	α chain and β chain	2025	https://tcr3d.ibbr.umd.edu
IEDB [163]	α chain and β chain	2018	https://www.iedb.org/
CEDAR [164]	α chain and β chain	2023	https://cedar.iedb.org/

Multiple sources provide different information on neoantigen prediction. For instance, neoantigens arising from RNA splicing are more likely to serve as shared neoantigens than those originating from SNVs [42]. It is crucial to explore additional neoantigen-related insights embedded within RNA sequencing (RNA-seq) datasets. Moreover, the diversity in the number of altered amino acids enhances the immunogenicity of neoantigens produced by alternative splicing. Consequently, it is essential to continue integrating advanced methodologies to discover more immunogenic neoantigens from different sources and expand the neoantigen database. Multi-omics analysis can be employed to integrate data from multiple dimensions, overcoming the limitations of single-omics approaches and providing a comprehensive understanding of neoantigen generation mechanisms.

### Comprehensive prediction algorithms

Numerous neoantigen prediction methods leveraging advanced models such as transfer learning, transformer, and GNN have been proposed in recent years. While these approaches have distinct advantages, the absence of a unified parameter reference standard leads to substantial performance variability across datasets and limited generalizability. This hinders their broader clinical applicability. Existing neoantigen data are often characterized by high dimensionality, sparsity, and heterogeneity, with significant differences in information across different cancer types. Consequently, there is a pressing need to develop universal prediction models capable of targeting a wider range of cancer types and accommodating diverse mutation profiles. Integrating state-of-the-art artificial intelligence algorithms offers the potential to extract additional insights. For instance, multi-omics methodologies can facilitate the elucidation of tumor-immune interactions. Incorporating TCR profiling, immune microenvironment data, and other relevant information into prediction models enables more holistic and accurate predictions. Currently, with the rapid application of multimodal approaches, their advantages in handling multi-dimensional feature datasets become evident. These models can accurately reflect the affinity information between peptides and HLA, as well as between TCR, thereby facilitating the prediction of neoantigens.

### General clinical applications

In the aspect of neoantigen prediction, the confirmation of immunogenicity represents a key step in the neoantigen discovery. Current approaches for assessing the immunogenicity of candidate peptides often rely on ELISpot assays and multicolor-labeled MHC tetramers. However, these conventional techniques possess notable limitations that necessitate the development of improved approaches. For instance, ELISpot assays may fail to detect low-affinity or non-secreting T cell populations, while MHC tetramer staining can miss functional T cells that exhibit low-affinity binding [184, 185]. To enhance efficiency, innovative methods should be developed, such as employing organoid models to replicate the tumor microenvironment, thereby enabling the validation of candidate neoantigens under more physiologically relevant conditions. Similarly, the validation of immunotherapeutic methods also has certain limitations. The number of cases in clinical trials for various tumor immunotherapies is relatively small. More comprehensive clinical trials, such as expanding the sample size and setting up more randomized controlled trials, can be conducted to increase the generalizability of the treatment methods.

Regarding universality, the generalizability of predicted neoantigens is a critical determinant of clinical immunotherapy treatment efficacy. In addition to differing in their origin, neoantigens can also be classified into two distinct categories: shared neoantigens and personalized neoantigens. Shared neoantigens represent mutations that are frequently observed among various cancer patients, while personalized neoantigens are specific to individual patients. Some cancer neoantigens arise from oncogene fragments with high mutational burden or genomic hotspot mutation regions. Mutant peptides generated from these regions and successfully presented to T cells can develop into shared neoantigens among different patients. For instance, PIK3CA, TP53, and KRAS, originating from driver gene mutations, have been identified in various cancer types, including colorectal cancer and melanoma [186]. The G12D mutation in KRAS is frequently observed in pancreatic cancer, colon adenocarcinoma, non-small cell lung cancer, and colorectal cancer. Singh et al. identified seven shared neoantigens, including KRAS-Q61H and TP53-R273N, that are commonly found across multiple pancreatic ductal adenocarcinoma patients [187]. Current research demonstrates that neoantigens arising from RNA splicing, non-coding region variations, and aberrant PTMs exhibit a higher likelihood of being shared across patients [188]. Targeting shared neoantigens offers a promising strategy to overcome the limitations associated with personalized therapies, particularly their inability to be mass-produced. This approach not only reduces costs of the design and application of therapeutic methods, but also enhances accessibility, enabling a broader population of cancer patients to benefit from these advancements. Private neoantigens represent patient-specific mutations that are unique to the individual and lack reproducibility across different patients. Utilizing private neoantigens as targets for immunotherapy significantly escalates both the temporal and financial costs associated with drug development. Consequently, current vaccine trials frequently employ a combination of multiple shared neoantigens to enhance efficiency and mitigate expenses.

In summary, while neoantigens hold significant promise for immunotherapy, additional in-depth research is essential to fully understand their characteristics and enhance clinical efficacy.

## Conclusion

Due to their tumor-specific properties, neoantigens have emerged as an increasing focus in cancer immunotherapy. Immunotherapeutic strategies targeting neoantigens can robustly activate immune responses and eliminate tumor cells. In this review, we delineated the primary sources of neoantigens currently identified, and systematically categorized them based on distinct classifications. Furthermore, we provided a comprehensive overview of the neoantigen prediction pipeline, compiling recent advancements in neoantigen prediction algorithms and explaining the technical strategy underpinning each method. Additionally, this paper highlights the various applications of neoantigens in immunotherapy and shows successful clinical cases. We also have collected the most recent databases related to neoantigens, as well as TCR sequence databases, to support subsequent research and experiments. Finally, we critically analyze the limitations and potential breakthroughs in neoantigen research, offering recommendations informed by the latest technological advancements.

## Data Availability

No datasets were generated or analysed during the current study.

## Abbreviations

A5SS: Alternative 5’ Splice Site
A3SS: Alternative 3’ Splice Site
ACT: Adoptive Cell Therapy
AML: Acute Myeloid Leukemia
APC: Antigen-Presenting Cell
AS: Ankylosing Spondylitis
CAR: Chimeric Antigen Receptor
CAR-T: Chimeric Antigen Receptor-engineered T cell
CDR3: Complementarity-Determining Region 3
CE: Cassette Exon
circRNA: circular RNA
CLL: Chronic Lymphocytic Leukemia
CNN: Convolutional Neural Network
CRC: Colorectal Cancer
CTA: Cancer-Testis Antigen
CV: Cancer Vaccine
DC: Dendritic Cell
dsRNA: double-stranded RNA
EL: Eluted Ligand
ELISlispot: Enzyme-Linked ImmunoSpot
FFNN: Feedforward Neural Network
FLC: Fibrolamellar Carcinoma
FSP: Frameshift Peptide
GBM: Glioblastoma
GC: Gastric Cancer
GNN: Graph Neural Network
HCC: Hepatocellular Carcinoma
HLA: Human Leukocyte Antigen
ICI: Immune Checkpoint Inhibitor
IR: Intron Retention
KNN: K-Nearest Neighbor
MHC: Major Histocompatibility Complex
MMR: Mismatch Repair
MSI: Microsatellite Instability
MSI-H: MSI-High
MXE: Mutually Exclusive Exon
NGS: Next-Generation Sequencing
NSCLC: Non-small Cell Lung Cancer
NLP: Natural Language Processing
ORF: Open Reading Frame
PBMC: Peripheral Blood Mononuclear Cell
pHLA: peptide-HLA
pMHC: peptide-MHC
PTM: Post-Translational Modification
INDEL: Insertion and Deletion
TAA: Tumor-Associated Antigen
TCR: T-Cell Receptor
TE: Transposable Element
TMB: Tumor Mutation Burden
TSA: Tumor-Specific Antigen
SNV: Single Nucleotide Variant
SV: Structural Variation
TCGA: The Cancer Genome Atlas
UTR: Untranslated Region
WES: Whole Exome Sequencing
WGS: Whole Genome Sequencing
